# Scaling Effect of Phosphorene Nanoribbon - Uncovering the Origin of Asymmetric Current Transport

**DOI:** 10.1038/srep38009

**Published:** 2016-11-29

**Authors:** Yawei Lv, Sheng Chang, Qijun Huang, Hao Wang, Jin He

**Affiliations:** 1 Department of Microelectronics, School of Physics and Technology, Wuhan University, Wuhan, Hubei 430072, PR China

## Abstract

In this paper, phosphorene nanoribbons (PNRs) are theoretically studied using a multiscale simulation flow from the ab initio level to the tight binding (TB) level. The scaling effects of both armchair PNRs (aPNRs) and zigzag PNRs (zPNRs) from material properties to device properties are explored. The much larger effective mass of holes compared to that of electrons in zPNR is responsible for its asymmetric transport. However, in aPNR, not only the effective mass difference but also the non-equal density of state (DOS) distributions near valence band maximum (VBM) and conduction band minimum (CBM) lead to the asymmetric transport. This non-equal distribution phenomenon is caused by energy band degeneracies near the VBM. Based on these two different mechanisms, PNRs’ asymmetric transport characteristics at the device level are explained, and it is shown that this behaviour can be ameliorated well by reducing the ribbon width in an aPNR MOSFET. Calculation results also indicate that aPNR’s effective mass is comparable to that of a graphene nanoribbon (GNR) at the same bandgap; however, aPNR’s band gap variation is more stable and regular than that of GNR, making it a good candidate for use in low-dimensional nano devices.

Two-dimensional materials such as monolayer graphene and MoS_2_ are extensively studied for the development of planar technology. Charge carriers in graphene are massless, which is favourable in current transport. However, its zero bandgap significantly hinders graphene’s applications. Although graphene can be cut into a nanoribbon to generate a bandgap, the effective mass also increases sharply with the scaling of the ribbon width^1^,^2^,^3^,^4^. As a naturally non-zero bandgap material, monolayer MoS_2_ is regarded as another competitive candidate for use in two-dimensional nano devices^5^,^6^,^7^,^8^. However, the low carrier mobility that makes it not so easy to support a large current is possibly the most serious problem of MoS_2 _9,10,11. Owing to its good balance between the bandgap and carrier mobility, phosphorene has attracted intense research interest^12^. The bandgap of monolayer phosphorene is approximately 1 eV and the reported carrier mobility is up to 1000 cm^2^/V · s^9^,^12^,^13^,^14^,^15^, making it suitable for quasi-two-dimensional applications. Anisotropic conducting behaviour is another interesting property of phosphorene due to its different effective masses of carriers along different transport directions^12^.

Following the continuous scaling down of nano devices, large area phosphorene is tailored into phosphorene nanoribbon (PNR)^16^,^17^,^18^,^19^, similar to graphene nanoribbon (GNR). PNR provides opportunities to use phosphorene in 1D nanoelectronics and to tune the electronic and transport properties of monolayer phosphorene^17^,^20^,^21^. Several studies have shown that the PNR bandgap increases with decreasing ribbon width and that PNR also shows asymmetric conducting behaviour^17^,^22^; several other properties such as the strain effect, edge saturation, defect, and stability have been studied as well^17^,^19^,^20^,^22^,^23^,^24^,^25^. However, the mechanism of asymmetric transport of electrons and holes in PNRs with different chiralities, which should be different from that of phosphorene because of PNR’s nano scale size, has not been elucidated.

The existing theoretical calculation methods of PNR face obstacles in the exploration of its transport mechanism. For examples, several studies utilized tight binding (TB) parameters of phosphorene^20^,^26^,^27^. Other researchers used computation theories such as the *k* · *p* d and effective mass methods^10^,^28^. However, in these methods, the key parameters are obtained from large area phosphorene, without considering the edge effect of PNR. Their availability and accuracy in PNR, especially when including the scaling effect, are limited.

Here, we introduce a new method for simulation of the material and transport properties of PNR. This simulation procedure has been verified for silicon and carbon devices, as reported in our previous works^29^,^30^. First, the edge effect is considered by a force relax calculation within the ab initio method. Then, the energy band properties are transformed perfectly into the TB parameters using the Wannier function^31^. These TB parameters obtained from the Wannier transformation describe PNR’s material properties more precisely than those obtained directly from large area phosphorene.

Using this method, both material properties and device properties of PNRs with different ribbon widths are simulated and summarized. We found that the hole current in armchair PNR (aPNR) is larger than the electron current, while in zigzag PNR (zPNR), the electron current is larger than the hole current, demonstrating the asymmetric transport in PNRs. To explore this interesting phenomenon, the energy band structures, densities of states (DOS) near the valence band minimum (VBM) and conduction band maximum (CBM), effective masses of electrons and holes, and respective field effect mobilities (*μ*_*FE*_) of PNR are analysed in detail. The mechanisms underlying aPNR’s and zPNR’s asymmetric transport behaviours are different, and as a scaling effect, the asymmetric behaviour of aPNR can be significantly ameliorated by reducing its ribbon width. The calculations also clearly show that the effective mass of aPNR is similar to that of aGNR at the same bandgap but that its bandgap is more stable and regular than that of GNR (which is helpful for reducing difficulties in technological implementation). Therefore, aPNR can be a competitive candidate for use in next-generation nano devices. Our explanation of the mechanism provides a theoretical foundation for its future applications.

## Results and Discussions

**Figure 1:**
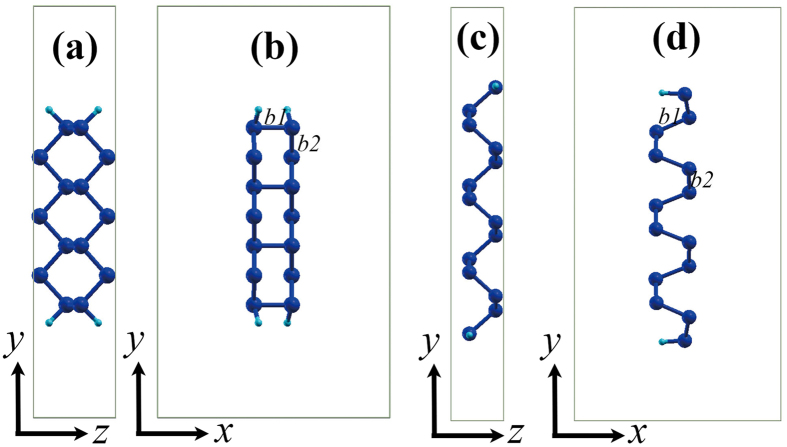
Front views (**a**,**c**) and side views (**b**,**d**) of 7-aPNR and 7-zPNR. Transport direction is along the *z* axis. P atoms are shown in deep blue, and they are saturated by H atoms, which are shown in light blue. Outside rectangles are the size of supercells.

**Figure 2:**
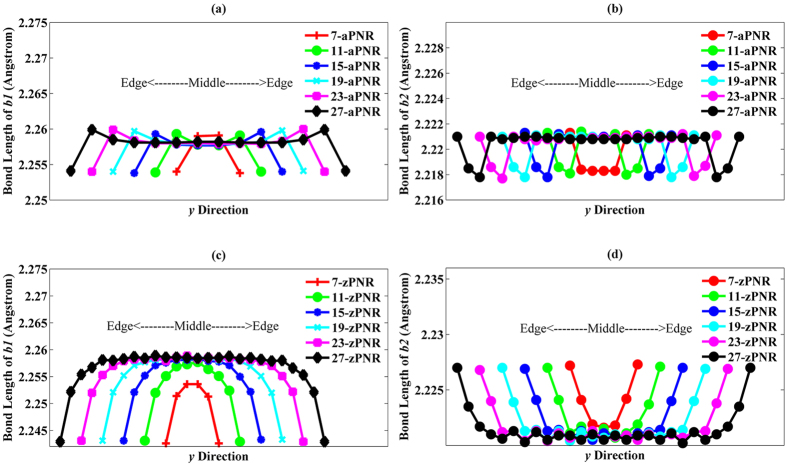
(**a**,**b**) Length variations of *b1* and *b2* in aPNRs. (**c**,**d**) Length variations of *b1* and *b2* in zPNRs. For better presentation, the middles of different ribbons are moved into the same *y* coordinate.

The supercells used as basic models in the ab initio simulation of PNRs are shown in Fig. 1. For convenience, PNRs are denoted by their widths (similar to GNRs). For example, the front views of 7-aPNR and 7-zPNR are shown in Fig. 1(a,c). In our calculations, PNRs are all saturated by H atoms drawn as light blue balls. The effect of substrate is not incorporated and a high pressure hydrogen environment is considered in order to eliminating reconstructions of PNR edges^32^,^33^. The rectangles outside are the sizes of the supercells and are set to be larger than the sizes of the PNR structures in both the *x* and *y* directions in order to remove the interactions with image PNRs in the ab initio computation. In the TB model that has been reported previously^34^, two types of bonds that induce the largest effect on the band structure of phosphorene are denoted as *b1* and *b2* in this figure. To release the forces, variable-cell calculation is adopted with the variations of these two types of bonds along with their positions shown in Fig. 2. It is clear that two or three bond lengths near edges show obvious variations. The edge effect induces increases in *b2* in zPNRs, while the edge bonds are reduced in other cases. Another interesting phenomenon is that the changes in *b2* at aPNR edges are less than those on the inside of the aPNR. The variation ranges of *b1* and *b2* in aPNR are approximately 0.005 Å, and the ranges in zPNR are 0.02 Å and 0.01 Å, respectively. Therefore, the edge reconstruction effect has a greater impact on zPNR than aPNR after force relaxation. Additionally, PNRs with narrower widths are more strongly impacted because of their higher fraction of the edge atoms. It can be concluded that the width scaling effect leads to changes in both aPNR and zPNR structures. Thus, it also can be inferred that their electronic properties are affected.

**Figure 3:**
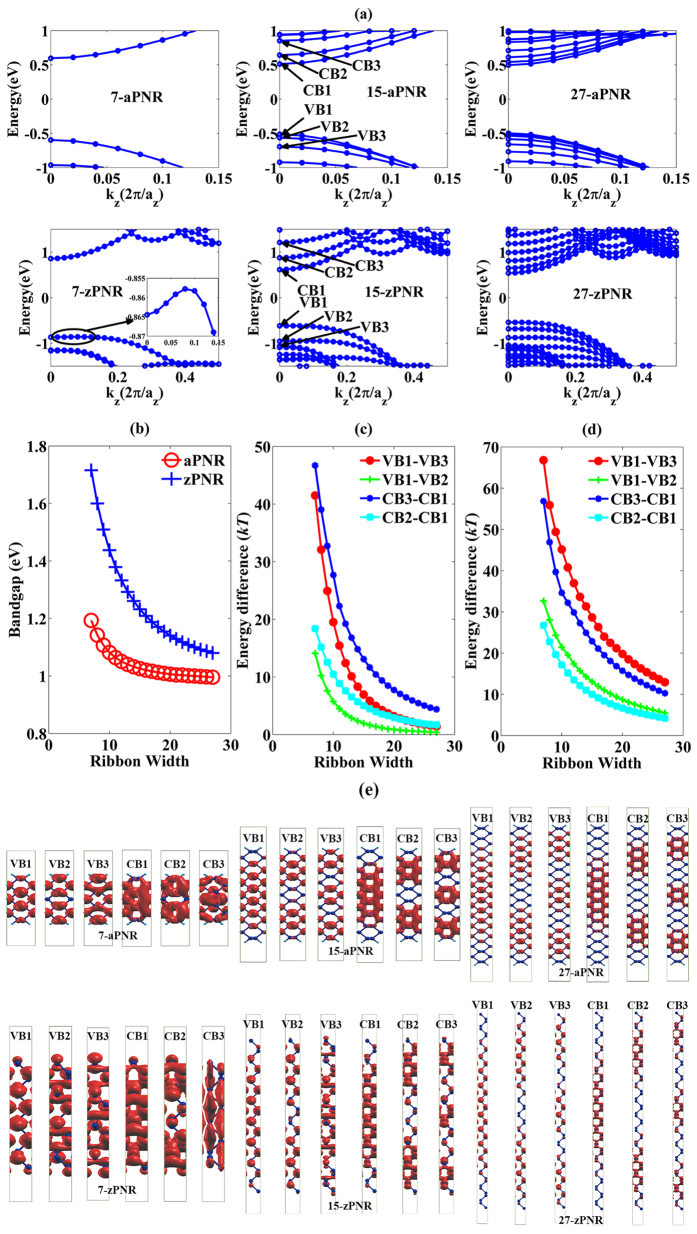
(**a**) Band structures of six types of PNRs. At Γ point, the three lowest conduction band valleys and three highest valence band valleys are denoted by CB1, CB2, CB3, and VB1, VB2, VB3 shown in 15-aPNR and 15-zPNR as examples. (**b**) Bandgap variations along with ribbon width. (**c**,**d**) Energy difference variations of energy valleys along with ribbon width in aPNR and zPNR, respectively. Real space distributions of energy valleys in different aPNRs and zPNRs are also shown in (**e**).**VB1, VB2,** ***etc***., are energy valleys in each PNR (shown in **(a)** as examples). Red colour shows the isosurface**s** of distribution probabilities of electrons within the respective valleys. The value of isosurfaces is 5 **× **10^−4^.

Following force relaxation, the band structures of PNRs with widths ranging from 7 to 27 were calculated. Figure 3(a) shows the results obtained for some PNRs as examples. The number of bands within the same energy range increases when the ribbon width is enlarged, and so do the energy valleys at Γ point. More importantly, these valleys tend to be more degenerate at CBM or VBM as the ribbon width increases. Because the band structures near the CBM and VBM are important for carrier transport, three conduction band valleys (CB1, CB2, and CB3) and three valence band valleys (VB1, VB2, and VB3) in every PNR are chosen and are discussed in detail. Energy differences of these valleys in aPNRs with different ribbon widths are shown in Fig. 3(c) in units of *kT*, where *k* is the Boltzmann’s constant and *T* is the absolute temperature (300 K in our calculation). It is clear that the energy band degeneracy appears at both valence and conduction bands in aPNRs when the ribbon widths are large, and the degeneracy of the valence bands increases much faster than that of the conduction bands. The energy difference between VB1 and VB2 drops significantly below 1 * *kT* when the ribbon width is larger than 19, and even the difference between VB1 and VB3 decreases rapidly. For CB1 and CB2, the lowest difference is approximately 1.6 * *kT*. Lower energy differences between VB1 & VB2 and VB1 & VB3 indicate a greater degeneracy of the valence bands that can induce a higher DOS at the VBM. On the other hand, degeneracy behaviour can be eliminated by reducing the ribbon width of the aPNR. Unlike aPNR, the energy differences of valleys in zPNRs are much larger as shown in Fig. 3(d). Although the tendency of decreasing energy differences can also be observed, decreasing velocities in conduction bands and valence bands are similar to each other and the lowest energy difference is still larger than 4 * *kT*, which means that no actual degeneracy exists in zPNRs. Thus, the DOS of CBM or VBM in zPNRs are mainly determined by the lowest conduction band or the highest valence band. Real space distributions of these energy valleys within aPNRs and zPNRs are displayed in Fig. 3(e), as a reference of energy band degeneracy. For aPNR with a wide ribbon width (27-aPNR), these energy states show the localization property. CB1 and VB1 are mainly located in the middle of the ribbon, which may be caused by the edge effect. It is interesting that CB2 and VB2 are forbidden in the middle of the PNR and that they are nearly complementary with CB3 and VB3, respectively. It can be deduced that the edge effect and band degeneracy jointly induce these localization phenomena, and the localizations within zPNRs are weaker than aPNRs because of the less degeneration tendency in the energy bands of zPNRs. As the ribbon width is scaled down, the localization behaviour is eliminated gradually and can hardly be observed in 7-aPNR. Weak localization in the narrow width PNR can help improve the transport properties in PNR since it can promote the transmission of carriers. However, this promotion effect may be limited considering other degeneration factors such as effective mass which will be discussed later.

In addition to energy band degeneracy, several other interesting phenomena can be seen in the bandgaps as shown in Fig. 3(b). The bandgaps and their range of variation of zPNRs are larger than those of aPNRs. In graphene, the hexagonal Brillouin zone is superimposed and touches the energy bands at the K-points. Six equivalent Dirac points are present at the boundary of graphene’s Brillouin zone, resulting in three families according to bandgap variation in its respective ribbons. However, inverse relations are observed between the ribbon widths and bandgaps in both aPNR and zPNR without being divided into different families because of the lack of equivalent Dirac points. Because the variation curves do not show any fluctuations, the PNR bandgaps are more regular and stable than those of GNRs. Moreover, the bandgaps in PNRs will still be maintained at values above that of the phosphorene bandgap (approximately 1 eV in our calculation) even when their widths are hugely enlarged. Thus, in device applications, PNRs can be much wider than GNRs which will undoubtedly reduce technological restrictions on their use and increase their current transport ability. It is also important to note the following subtle detail: as their width is scaled down, aPNRs remain direct-gap semiconductors; however, in zPNR, the VBM begins to shift away from the Γ point for widths less than 17, making zPNRs indirect-gap-like semiconductors. As illustrated in the plot of 7-zPNR shown in the insert of Fig. 3(a), the VBM is located at 0.08 * *k*_*z*_ and the energy difference between the VBM and the highest energy at the Γ point is 7 meV. This characteristic may influence the variation of the zPNR hole effective mass, as discussed below.

**Figure 4:**
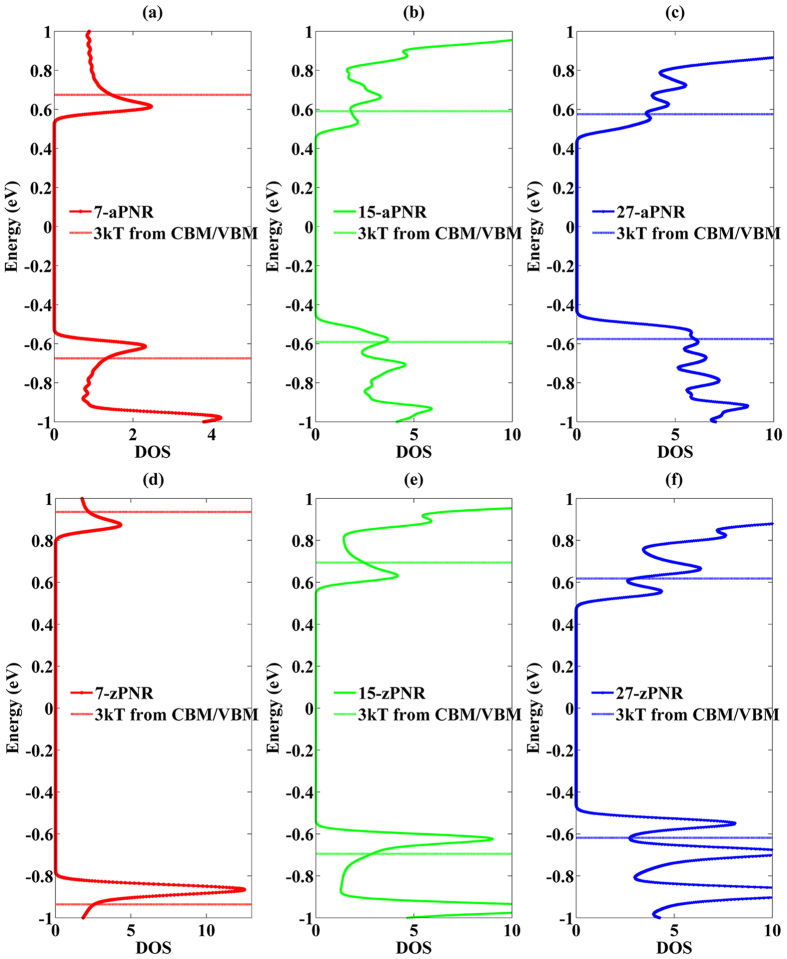
DOS of several aPNRs (**a**,**b**,**c**) and zPNRs (**d**,**e**,**f**). The horizon lines are 3 * *kT* away from CBM or VBM.

As an important factor in carrier transport, DOS (depicted in Fig. 4) verifies the effect of energy band degeneracy in PNRs. The horizon lines are 3 * *kT* away from the CBM or VBM. In 7-aPNR, the DOS near the CBM and VBM are similar to each other because the energy band degeneracies are not obvious. However, with the increase in the ribbon width, DOS are increased as well because VB2 and VB3 move upward and CB2 and CB3 move downward (Fig. 3(a)). The degeneracy appears more rapidly for the valence bands than for the conduction bands. Thus, DOS near the VBM become higher than the CBM when the ribbon width is increased, as seen for 15-aPNR and 27-aPNR for which the VBM DOS are almost twice as large as the CBM DOS. In 27-aPNR, the DOS peaks which are induced by the overlap of individual energy valley provides another proof of the existence of energy band degeneracies.

For a comparison, the zPNR DOS are also shown. It is clear that the DOS peaks induced by each energy valley can be clearly distinguished even when the ribbon width reaches 27 because there is no obvious energy band degeneracy, as discussed above. However, the VBM DOS are still higher than the CBM DOS. It is inferred from the above analyses that the high DOS of VBM in aPNRs are caused by band degeneracies, while in zPNRs, DOS due to the highest valence band are higher than due to the lowest conduction band.

**Figure 5:**
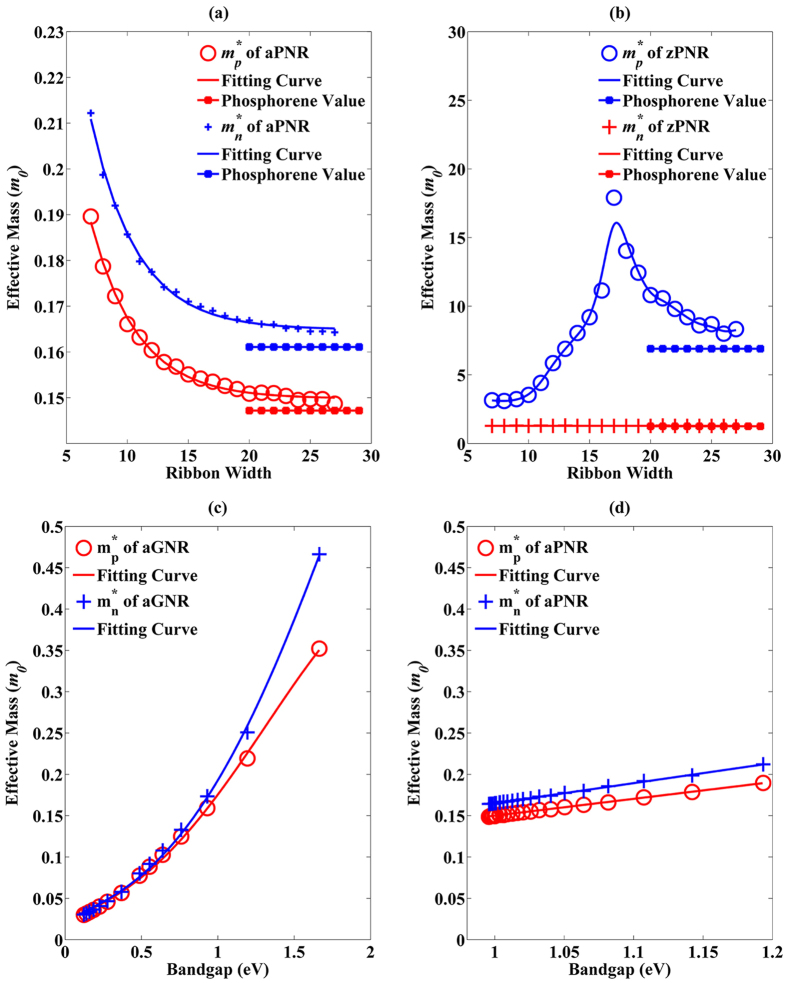
(**a**,**b**) Effective mass variations along with ribbon width in aPNRs and zPNRs, respectively. To compare to GNRs, effective mass variations together with bandgaps in aGNRs and aPNRs are also shown in (**c**,**d**), respectively.

The role of effective masses near the CBM and VBM is explored, as shown in Fig. 5. Figure 5(a) shows that effective masses of both electrons (*m*_n_*) and holes (*m*_p_*) of aPNRs increase with the scaling down of ribbon width and show a nearly exponential distribution. Within the variation, *m*_n_* is 10% larger than *m*_p_* up to their phosphorene values of 0.16*m_*0*_ for *m*_n_* and 0.15*m_*0*_ for *m*_p_*, where *m*_*0*_ is the absolute mass of an electron in a vacuum. This small difference may not fully account for asymmetric transport in aPNRs, especially when the ribbon width is large. Fitting equations for hole (1) and electron (2) effective masses of aPNRs can be sorted as


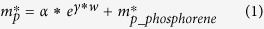


and


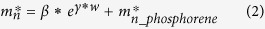


where *α* and *β* are fitting coefficients equal to 0.2415 and 0.2887, respectively. The exponential coefficient *γ* is 0.262 for both *m*_p_* and *m*_n_*, proving that holes and electrons have similar variation tendencies. *w* is the ribbon width in the unit of atoms. 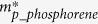 and 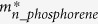 can be treated as effective masses of holes and electrons in phosphorene along the armchair direction irrespective of slight deviations and are equal to 0.1498 and 0.1649, respectively. It is noted that after the ribbon width is reduced from 27 to 7, only a 25% increase in the effective mass is observed in aPNR; this means that the ribbon width of aPNR can be scaled down without a significant loss of transport ability.

Unlike in aPNRs, the effective masses in zPNRs are much larger, as can be observed in Fig. 5(b). *m*_n_* maintains its phosphorene value (1.2 * *m*_*0*_) with the reduction of the ribbon width, while *m*_p_* first increases but then decreases when the ribbon width is less than 17. This is because the VBM moves away from the Γ point and becomes similar to an indirect gap semiconductor, as discussed above, with an increase in the VBM curvature after the shift. The minimum value of *m*_p_* is 3.2 * *m*_*0*_, which is still much larger than *m*_n_*, whereas its phosphorene value is 6.9 * *m*_*0*_. The high DOS induced by the highest valence band in zPNRs shown in Fig. 4(d–f) can also be attributed to the large effective masses. However, this property can hardly induce hole currents that are larger than electron currents because *m*_p_* in zPNRs are too large.

For a meaningful comparison, the effective mass variations along with bandgaps of aGNRs and aPNRs are drawn together in Fig. 5(c,d). Although both *m*_n_* and *m*_p_* in aGNR are very low when the bandgap is small, they increase quickly when the bandgap is enlarged, with the increasing trend obeying Gaussian fitting. This means that the bandgap enlarges the aGNR effective mass because the Dirac point is broken^2^,^11^,^35^. In contrast, the increases in *m*_n_* and *m*_p_* in aPNRs are much lower and they obey linear fitting with small slopes. When comparing the effective masses of aGNRs and aPNRs at the same bandgap, the low effective mass advantage no longer exists in aGNRs. The effective masses of zGNRs and zPNRs are not compared because zGNRs are gapless.

**Figure 6:**
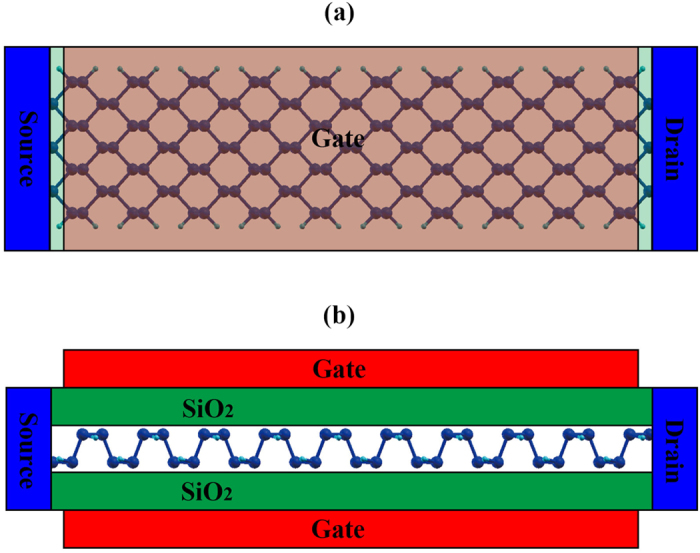
Front view (**a**) and side view (**b**) of a PNR MOSFET model with double-gate structure.

In addition to the material parameter analysis, the asymmetric transport in PNRs can be directly demonstrated in device-level simulations. In our procedure, TB Hamiltonians of PNRs are obtained using maximally localized Wannier functions (MLWF)^31^,^36^. Because of the MLWFs’ unitary transformation property, the material properties of PNRs are accurately represented by the TB parameters. Using these TB parameters, Schrodinger’s equation is solved by the non-equilibrium Green’s function (NEGF) method and the final transport properties are obtained by iterations between Schrodinger’s and Poisson’s equations. To compare the current properties of different PNRs at the device level, a PNR MOSFET model is constructed and is shown in Fig. 6. A double-gate structure is chosen for its good performance in nano device design. The SiO_2_ layers on both sides are 1-nm thick. The channel length is 10 nm and it is undoped. The channel is connected to virtual reservoirs at its ends acting as the source and drain^37^. Carriers within the channel are assumed to be ballistic transport since the channel length is only 10 nm. Both the source and drain are assumed to be heavily doped and their Fermi levels only can be controlled by the source to drain voltage. At zero gate voltage (*V*_*g*_), the Fermi level of the channel is kept in the middle of the PNR’s forbidden band by a translation of the diagonal terms in the Hamiltonian. The source voltage is kept at zero, and the source to drain voltage (*V*_*d*_) is 0.1 V.

**Figure 7:**
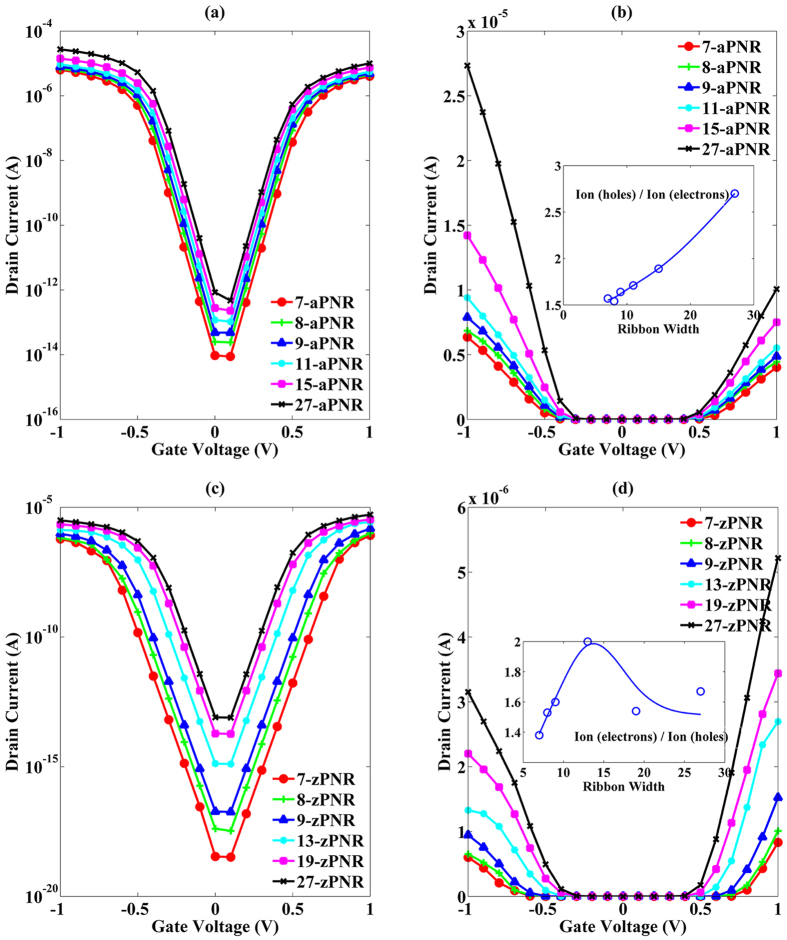
(**a**,**b**) Transfer curves of aPNR MOSFETs in logarithmic and linear scales. (**c**,**d**) Transfer curves of zPNR MOSFETs in logarithmic and linear scales. Inset plots in (**b**,**d**) are ratios of *I*_*on*_ contributed by different carriers. All curves are calculated at *V*_*d*_ = 0.1 V, and *I*_*on*_ is defined as the drain current at *V*_*g*_ = ±1 V.

Transfer curves of MOSFETs with different PNR channels are presented in Fig. 7. As shown in Fig. 7(a), as the ribbon width is increased, both on (*I*_*on*_) and off (*I*_*off*_) state currents are increased. However, *I*_*off*_ of all aPNR MOSFETs are still maintained below 10^−6^ mA, which is a tiny value in digital logical circuits, and *I*_*on*_ are several or dozens of mA, as seen from the linear scale plots in Fig. 7(b). It is also noted that the asymmetric current transports in aPNR MOSFETs are significant and that the ratios between the hole and electron currents increase with increasing ribbon width (seen in the inserted plot). When the ribbon width is 7, the ratio between the hole and electron currents is approximately 1.5 and can be treated as induced by the effective mass difference. Both *I*_*on*_ and *I*_*off*_ reach several mA, which can meet the current requirement for low-dimensional devices. With increasing ribbon width, the current ratio is increased and reaches 2.7 at the 27-aPNR MOSFET. Because in this case, *m*_p_* is maintained at just approximately 10% lower than *m*_n_* as shown in Fig. 4(a), the enhancement of the current ratio is mainly caused by the rapidly rising degeneracy of the valence bands in aPNRs. On the other hand, *I*_*off*_ in zPNR MOSFETs are smaller as shown in Fig. 7(c) due to their wider bandgaps. However, their *I*_*on*_ are also about one order of magnitude lower than those of aPNR MOSFETs as shown in Fig. 7(d) because they have much larger effective masses of both holes and electrons. zPNR’s electron currents are much higher than their hole currents because the effective mass differences between electrons and holes are significant, dominating the current ratio between electrons and holes. This is demonstrated by the similarity of the current ratio variation tendency in the inset of Fig. 7(d) to that of the effective mass ratios of holes and electrons shown in Fig. 5(d). The subthreshold slopes (*SS*) of both aPNT MOSFETs and zPNR MOSFETs are all near 60 mV/dec when the gate voltage is low because of the assumption of ballistic transport. However, the *SS* in zPNR MOSFETs are degenerated rapidly with the increasing of gate voltage, which also means an unsuitable property in current transport.

**Figure 8:**
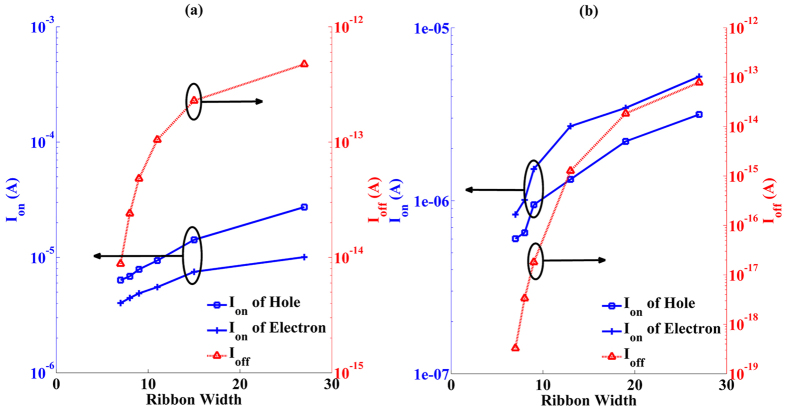
*I*_*on*_ and *I*_*off*_ variations of aPNR MOSFETs (**a**) and zPNR MOSFETs (**b**) along with ribbon widths. Curves and *y* coordinates shown in the same colour correspond to each other.

The *I*_*on*_ and *I*_*off*_ of PNR MOSFETs are presented in Fig. 8. It is clear that with increasing ribbon width, *I*_*off*_ of both aPNR and zPNR MOSFETs first increase rapidly and then flatten out. This is consistent with their bandgap variations as a key factor in the modulation of *I*_*off*_. Larger bandgaps induce lower currents in the off state, and therefore, *I*_*off*_ in zPNR MOSFETs are lower than in the aPNR MOSFETs. In addition to *I*_*off*_, *I*_*on*_ are also affected by the bandgaps due to the shifts in the threshold voltage (*V*_*th*_). Thus, the increasing tendencies of *I*_*on*_ in aPNR MOSFETs and zPNR MOSFETs are all similar to that of *I*_*off*_ but with different slopes. Based on the above analyses, it can be concluded that aPNR is more favourable for use in MOSFET because of its stronger current transport ability. When cut into narrower ribbon, its asymmetric behaviour can be relieved without significant loss current transport ability because of its stable effective mass variation and delocalized energy state distribution when the width is scaled down.

Based on the channel currents of PNR MOSFETs, *μ*_*FE*_ of carriers can be computed according to equation (3)^9^, which also demonstrates the role of the effective mass in current transport


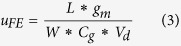


where *L* and *W* are the length and width of the PNR MOSFET, *V*_*d*_ is the source to drain voltage (0.1 V in our calculation), and *g*_*m*_ is the transconductance. To obtain more precise mobility values, gate capacitance (*C*_*g*_) is calculated as *dQ*_*channel*_/*dV*_*g*_, where *Q*_*channel*_ are the charges in the channel and *V*_*g*_ is the gate voltage that can be regarded as the total serial capacitance of the oxide capacitance and quantum capacitance. For verification of the influence of the effective mass on current transport, the final *μ*_*FE*_ values are summarized in Table 1. In aPNR MOSFETs, *μ*_*FE*_ are more than 100 cm^2^/V · s, similar to Si and GNR MOSFETs at the same bandgap. The differences between electron’s *μ*_*FE*_ and hole’s *μ*_*FE*_ are approximately 10%, which is consistent with their effective mass values. Because aPNR MOSFETs’ differences in *μ*_*FE*_ or effective masses are insufficient for inducing such large asymmetric I_on_ (for example, the ratio between the hole and electron currents is 2.7 in 27-aPNR MOSFET), this shows that the differences between the hole and electron currents in aPNR MOSFETs are caused not only by the holes’ slightly small effective masses but more importantly by the larger DOS in the VBM induced by energy band degeneracy, especially for wider ribbon widths. On the other side, the mobilities of zPNR MOSFETs are much lower than those of the aPNR MOSFETs because of their larger effective masses. Furthermore, *μ*_*FE*_ of electrons are approximately two times larger than those for the holes in zPNR MOSFETs, directly leading to larger electron currents.

## Conclusions

In this paper, the scaling effect of PNRs and their corresponding devices are studied using a multiscale simulation procedure. Unlike the semi-empirical TB or *k* · *p* methods, TB parameters in this procedure are obtained by unitary transformations from ab initio Bloch wave functions to TB orbitals. Therefore, the edge effects of PNRs computed using ab initio methods can be incorporated into their TB Hamiltonians and are reflected in the device-level transport properties. Simulations reveal that asymmetric transport behaviour exists in both aPNR and zPNR devices. Hole currents in aPNRs are larger due to their small *m*_p_* at the VBM and faster valence band degenerations, whereas electron currents are larger in zPNRs because their *m*_n_* are much smaller than *m*_p_*. The currents in aPNR MOSFETs are one order of magnitude larger than in zPNR MOSFETs, indicating that aPNRs are more appropriate in MOSFET application and that the asymmetric current transport behaviour can be ameliorated when the ribbon width is small. Comparison of aPNRs and aGNRs shows that their effective masses and field effect mobilities are similar. Furthermore, PNRs can be used in a much wider range of applications than GNRs because of the natural bandgap in phosphorene and because the bandgap variation in aPNR is more stable and regular than in aGNR. All of the above the properties make aPNR a competitive candidate for use in low-dimensional nano devices. Furthermore, our explanation of the mechanism provides a theoretical foundation for the future applications of PNR.

### Simulation Methods

A multiscale simulation procedure is adopted in this simulation study^29^,^30^,^38^,^39^. First, ab initio density functional theory (DFT) is used as implemented within the QUANTUM ESPRESSO open source package^40^. To obtain a stable PNR structure with the forces relaxed, variable-cell calculation with Perdew-Burke-Ernzerhof (PBE) exchange-correlation functional is used. The total energy change between two consecutive self-consistent field (SCF) steps is less than 1 × 10^−4^ Ry, and all components of all forces are smaller than 1 × 10^−4^ Ry/a.u. The total energy criterion within one SCF calculation is 1 × 10^−12^ Ry^11^,^12^. Then, in order to avoid the underestimation of the PNR’s bandgap^11^,^41^, the PBE and VDW-DF functionals are used together in the band structure calculation step. In our calculations, the bandgaps of monolayer and multilayer phosphorenes are 0.3 eV and 1 eV, consistent with the reported experimental values^14^. The pseudopotential is ultrasoft, and the kinetic energy cutoff is 40 Ry^17^,^42^. A vacuum spacing of more than 10 Å is used to separate the GNR from its images resulting from the periodic boundary condition. Although several studies used larger spacing in their calculations^34^, we have tested that 10 Å is sufficient to obtain electronic properties that are exactly the same as those obtained using a space of 20 Å. Two 1 × 1 × 10 and 1 × 1 × 25 *k*-meshes are used in SCF and non-SCF calculations, respectively^11^,^13^.

To obtain the final transport property, Wannier transformation is then adopted as implemented in the wannier90 open source code^43^. The electron wavefunctions computed in ab initio calculations are transformed into the TB form, and the TB Hamiltonian is also obtained. The traditional 1 × 1 × 25 *k*-mesh is not sufficient for computing DOS. This problem can be overcome by Wannier interpolations, and more accurate DOS calculations are achieved. Finally, the TB Hamiltonian is used by another open source code, NanoTCAD ViDES^44^, devoted to solving the transport problem at the nano scale using the non-equilibrium Green’s function (NEGF) method^45^. Computational details are mainly focused on the iterations between Schrodinger’s equation and Poisson’s equation. Benefiting from the verified and reliable TB Hamiltonian obtained from a unitary transformation, the PNR’s material properties can be preserved and are reflected in the transport properties of the respective devices.

## Additional Information

**How to cite this article**: Lv, Y. *et al*. Scaling Effect of Phosphorene Nanoribbon - Uncovering the Origin of Asymmetric Current Transport. *Sci. Rep.* **6**, 38009; doi: 10.1038/srep38009 (2016).

**Publisher's note:** Springer Nature remains neutral with regard to jurisdictional claims in published maps and institutional affiliations.

## Figures and Tables

**Table 1:** Field effect carrier mobilities in PNR MOSFETs (cm^2^/V·s).

Type	aPNR MOSFETs						zPNR MOSFETs					
Width	7	8	9	11	15	27	7	8	9	13	19	27
Hole Mobility	113	137	131	123	147	156	12	9	10	9	9	16
Electron Mobility	105	109	121	113	123	135	26	24	28	27	27	28

## References

[b1] Schwierz F. Graphene transistors. Nat. Nanotechnol. 5, 487–496 (2010). 20512128 10.1038/nnano.2010.89

[b2] Novoselov K. S. . Two-dimensional gas of massless dirac fermions in graphene. Nature 438, 197–200 (2005). 16281030 10.1038/nature04233

[b3] Chen Y.-C. . Tuning the band gap of graphene nanoribbons synthesized from molecular precursors. ACS Nano 7, 6123–6128 (2013). 23746141 10.1021/nn401948e

[b4] Xia F., Farmer D. B., Lin Y. M. & Avouris P. Graphene field-effect transistors with high on/off current ratio and large transport band gap at room temperature. Nano Lett. 10, 715–718 (2010). 20092332 10.1021/nl9039636

[b5] Radisavljevic B., Radenovic A., Brivio J., Giacometti V. & Kis A. Single-layer MoS_2_ transistors. Nat. Nanotechnol. 6, 147–150 (2011). 21278752 10.1038/nnano.2010.279

[b6] Zhang Y., Ye J., Matsuhashi Y. & Iwasa Y. Ambipolar MoS_2_ thin flake transistors. Nano Lett. 12, 1136–1140 (2012). 22276648 10.1021/nl2021575

[b7] Das S., Chen H. Y., Penumatcha A. V. & Appenzeller J. High performance multilayer MoS_2_ transistors with scandium contacts. Nano Lett. 13, 100–105 (2013). 23240655 10.1021/nl303583v

[b8] Yoon Y., Ganapathi K. & Salahuddin S. How good can monolayer MoS_2_ transistors be? Nano Lett. 11, 3768–3773 (2011). 21790188 10.1021/nl2018178

[b9] Das S., Demarteau M. & Roelofs A. Ambipolar phosphorene field effect transistor. ACS Nano 8, 11730–11738 (2014). 25329532 10.1021/nn505868h

[b10] Haratipour N., Namgung S., Oh S. H. & Koester S. J. Fundamental limits on the subthreshold slope in schottky source/drain black phosphorus field-effect transistors. ACS Nano 10, 3791–3800 (2016). 26914179 10.1021/acsnano.6b00482

[b11] Dai J. & Zeng X. C. Bilayer phosphorene: Effect of stacking order on bandgap and its potential applications in thin-film solar cells. J. Phys. Chem. Lett. 5, 1289–1293 (2014). 26274486 10.1021/jz500409m

[b12] Liu H. . Phosphorene: An unexplored 2d semiconductor with a high hole mobility. ACS Nano 8, 4033–4041 (2014). 24655084 10.1021/nn501226z

[b13] Peng X., Wei Q. & Copple A. Strain-engineered direct-indirect band gap transition and its mechanism in two-dimensional phosphorene. Phys. Rev. B 90, 085402 (2014).

[b14] Das S. . Tunable transport gap in phosphorene. Nano Lett. 14, 5733–5739 (2014). 25111042 10.1021/nl5025535

[b15] Rodin A. S., Carvalho A. & Castro Neto A. H. Strain-induced gap modification in black phosphorus. Phys. Rev. Lett. 112, 176801 (2014). 24836264 10.1103/PhysRevLett.112.176801

[b16] Carvalho A., Rodin A. S. & Castro Neto A. H. Phosphorene nanoribbons. Europhys. Lett. 108, 47005 (2014).

[b17] Han X., Stewart H. M., Shevlin S. A., Catlow C. R. & Guo Z. X. Strain and orientation modulated bandgaps and effective masses of phosphorene nanoribbons. Nano Lett. 14, 4607–4614 (2014). 24992160 10.1021/nl501658d

[b18] Tran V. & Yang L. Scaling laws for the band gap and optical response of phosphorene nanoribbons. Phys. Rev. B 89, 245407 (2014).

[b19] Guo H., Lu N., Dai J., Wu X. & Zeng X. C. Phosphorene nanoribbons, phosphorus nanotubes, and van der waals multilayers. J. Phys. Chem. C 118, 14051–14059 (2014).

[b20] Poljak M. & Suligoj T. Immunity of electronic and transport properties of phosphorene nanoribbons to edge defects. Nano Res. 9, 1723–1734 (2016).

[b21] Masih Das P. . Controlled sculpture of black phosphorus nanoribbons. ACS Nano 10, 5687–5695 (2016). 27192448 10.1021/acsnano.6b02435 PMC5897108

[b22] Peng X., Copple A. & Wei Q. Edge effects on the electronic properties of phosphorene nanoribbons. J. Appl. Phys. 116, 144301 (2014).

[b23] Xie J., Si M. S., Yang D. Z., Zhang Z. Y. & Xue D. S. A theoretical study of blue phosphorene nanoribbons based on first-principles calculations. J. Appl. Phys. 116, 073704 (2014).

[b24] Li W., Zhang G. & Zhang Y.-W. Electronic properties of edge-hydrogenated phosphorene nanoribbons: A first-principles study. J. Phys. Chem. C 118, 22368–22372 (2014).

[b25] Zhang X. . Tuning carrier mobility of phosphorene nanoribbons by edge passivation and strain. Phys. Lett. A 380, 614–620 (2016).

[b26] Yin D., Han G. & Yoon Y. Scaling limit of bilayer phosphorene fets. IEEE Electron Device Lett. 36, 978–980 (2015).

[b27] Ezawa M. Topological origin of quasi-flat edge band in phosphorene. New J. Phys. 16, 115004 (2014).

[b28] Wan R., Cao X. & Guo J. Simulation of phosphorene schottky-barrier transistors. Appl. Phys. Lett. 105, 163511 (2014).

[b29] Lv Y., Chang S., Wang H., He J. & Huang Q. Energy gap tunable graphene antidot nanoribbon MOSFET: A uniform multiscale analysis from band structure to transport properties. Carbon 101, 143–151 (2016).

[b30] Lv Y., Wang H., Chang S., He J. & Huang Q. Band structure effects in extremely scaled silicon nanowire MOSFETs with different cross section shapes. IEEE Trans. Electron Devices 62, 3547–3553 (2015).

[b31] Marzari N., Mostofi A. A., Yates J. R., Souza I. & Vanderbilt D. Maximally localized wannier functions: Theory and applications. Rev. Mod. Phys. 84, 1419–1475 (2012).

[b32] Gao J., Zhang G. & Zhang Y. W. The critical role of substrate in stabilizing phosphorene nanoflake: A theoretical exploration. J. Am. Chem. Soc. 138, 4763–4771 (2016). 27022974 10.1021/jacs.5b12472

[b33] Gao J., Liu X., Zhang G. & Zhang Y.-W. Nanotube-terminated zigzag edges of phosphorene formed by self-rolling reconstruction. Nanoscale ; doi: 10.1039/c6nr06201f (2016). 27725985

[b34] Rudenko A. N. & Katsnelson M. I. Quasiparticle band structure and tight-binding model for single- and bilayer black phosphorus. Phys. Rev. B 89, 201408 (2014).

[b35] Son Y.-W., Cohen M. L. & Louie S. G. Energy gaps in graphene nanoribbons. Phys. Rev. Lett. 97, 216803 (2006). 17155765 10.1103/PhysRevLett.97.216803

[b36] Calzolari A., Marzari N., Souza I. & Buongiorno Nardelli M. Ab initio transport properties of nanostructures from maximally localized wannier functions. Phys. Rev. B 69, 035108 (2004).

[b37] Datta S. Nanoscale device modeling: The green’s function method. Superlattices Microstruct. 28, 253–278 (2000).

[b38] Bruzzone S., Iannaccone G., Marzari N. & Fiori G. An open-source multiscale framework for the simulation of nanoscale devices. IEEE Trans. Electron Devices 61, 48–53 (2014).

[b39] Brandbyge M., Mozos J.-L., Ordejón P., Taylor J. & Stokbro K. Density-functional method for nonequilibrium electron transport. Phys. Rev. B 65, 165401 (2002).

[b40] Giannozzi P. . Quantum espresso: A modular and open-source software project for quantum simulations of materials. J. Phys.: Condens. Matter 21, 395502 (2009). 21832390 10.1088/0953-8984/21/39/395502

[b41] Qiao J., Kong X., Hu Z.-X., Yang F. & Ji W. High-mobility transport anisotropy and linear dichroism in few-layer black phosphorus. Nat. Commun. 5, 4475 (2014). 25042376 10.1038/ncomms5475 PMC4109013

[b42] Wei Q. & Peng X. Superior mechanical flexibility of phosphorene and few-layer black phosphorus. Appl. Phys. Lett. 104, 251915 (2014).

[b43] Mostofi A. A. . Wannier90: A tool for obtaining maximally-localised wannier functions. Comput. Phys. Commun. 178, 685–699 (2008).

[b44] Fiori G. & Iannaccone G. NanoTCAD ViDES. http://vides.nanotcad.com/vides (2005) (Date of access:15/10/2013).

[b45] Fiori G. & Iannaccone G. Multiscale modeling for graphene-based nanoscale transistors. Proc. IEEE 101, 1653–1669 (2013).

